# Continuous Near-Field Electrospraying Using a Glass Capillary Nozzle

**DOI:** 10.3390/mi9020056

**Published:** 2018-01-31

**Authors:** Xiang Wang, Jinghua Lin, Jiaxin Jiang, Shumin Guo, Wenwang Li, Gaofeng Zheng

**Affiliations:** 1 School of Mechanical and Automotive Engineering, Xiamen University of Technology, Xiamen 361024, China; wx@xmut.edu.cn (X.W.); ljh@stu.xmut.edu.cn (J.L.); 2 Department of Instrumental and Electrical Engineering, Xiamen University, Xiamen 361005, China; jiangjx@xmu.edu.cn; 3 Xiamen Key Laboratory of Optoelectronic Transducer Technology, Xiamen 361005, China; 4 Fujian Key Laboratory of Universities and Colleges for Transducer Technology, Xiamen 361005, China; 5 School of Mathematical Sciences, Xiamen University, Xiamen 361005, China; shumin_guo@xmu.edu.cn

**Keywords:** near-field, electrospray, printing, drop-on-demand, jetting

## Abstract

A continuous near-field electrospray process has been developed to deposit micropatterns. Different from traditional electrospray technologies, the nozzle-to-substrate distance was shortened to less than 5 mm, and a glass capillary nozzle with a diameter of tens of microns was used. Steady and continuous ejection was achieved, and patterns with line widths of sub-100 μm were generated. The influence of experimental parameters was investigated. The critical voltage for electrospray increased with nozzle-to-substrate distance and flow rate. The line width of electrosprayed patterns increased with the increases in applied voltage, flow rate, nozzle diameter, and deposition time. This work provides a simple and potential route for on-demand deposition of micro-/nano-patterns in the electrospray process.

## 1. Introduction

Electrospraying [1,2] is an emerging technology that utilizes an electric field to realize liquid atomization to fabricate micro-/nanometer-scale droplets, particles, and thin films and is now considered a versatile tool to deposit fluidic materials, particularly polymers and biomaterials for various applications such as nano-devices [3], biomedicine [4], sensors [5], and energy storage [6,7]. There are also many techniques for the fabrication of nano-/micro-particles and thin films, including pulsed laser deposition, magneton sputtering, and plasma spraying. Compared with these methods, the electrospray process demonstrates advantages such as the feasibility to conduct functional patterns with simple equipment, a low cost, and good material compatibility. The significant and fundamental difference between electrospraying and other widespread commercial technologies is the principle of droplet formation. In the electrospray process, liquid ejecting from the spinneret is induced by the charges that are activated by an applied electric field and accumulated on the liquid-air surface. The charged liquid, which is subjected to an electrostatic force under the electric field, will eject a thread when the electrostatic force overcomes the surface tension force. Subsequently, the liquid thread is atomized due to the Columbic interaction of charges under that electric field. The electrosprayed particles can range from several tens of nanometers to hundreds of micrometers with monodisperse distribution.

In the traditional electrospray setup, the distance from the nozzle to the substrate is usually greater than tens of centimeters, and the electrosprayed area on the substrate is relatively large. Thus, traditional electrospraying is suitable for preparing large atomized particles or films. In order to generate micro-/nano-patterns in the electrospray process, various template/molding strategies are usually utilized. For example, Nithyanandan et al. [8] used template-assisted electrospraying to deposit line arrays with widths of about 50 μm. Zhu et al. [9] used a template-assisted method to fabricate micropatterns of a nano-hydroxyapatite/silk fibroin composite. Higashi et al. [10] generated micropatterns of silica nanoparticles via electrospray deposition with a stencil mask. Xie et al. [11] achieved precise particle patterns and cell patterns via electric-field-controlled electrospray deposition. However, these methods require elaborate operations, making them undesirable for practical applications.

Direct writing [12] is a maskless, flexible, and multi-length-scale process for the deposition of functional and structural materials on a substrate. Patterns of simple lines or complex structures can be deposited directly by controlling the movement of the substrate. Experiments combining direct writing and electrospray technology have been carried out using short nozzle-to-substrate distances and probe spinnerets and have shown the feasibility of controllable electrospraying for the microscale deposition of atomized particles [13,14]. However, the solution feeding approach in these processes limits the total length of particle deposition, and the line widths are non-uniform because the solution is consumed in the process.

In this paper, we propose continuous near-field electrospraying where the nozzle-to-substrate distance is shortened to 0.5–5 mm and a tiny glass capillary nozzle is used. Long-term continuous electrospraying in a small area was achieved. In addition, the effects of the experimental parameters were investigated, and complex patterns were obtained.

## 2. Materials and Methods

**Figure 1:**
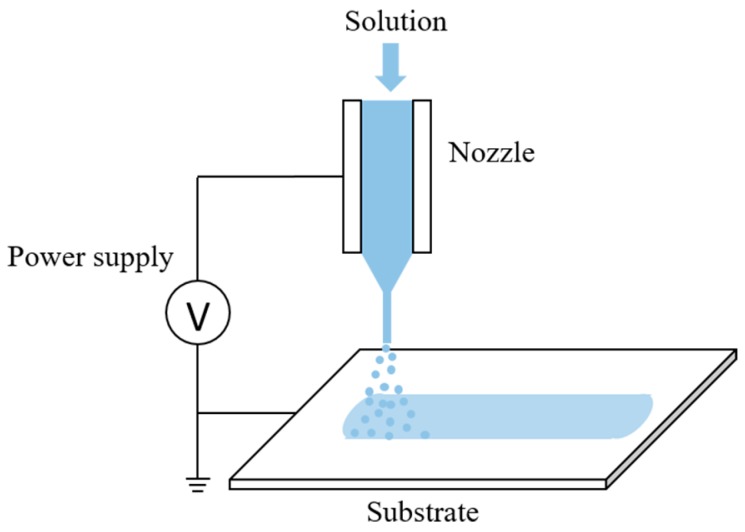
Schematic of experimental setup.

The experimental setup is schematically shown in Figure 1 and includes a high voltage power supply, a nozzle, a substrate, and a precise syringe pump. The high voltage power supply (DW-P403-1AC, Tianjin Dongwen High Voltage Power Supply Plant, Tianjin, China) provided a potential between the nozzle and the grounded copper substrate. The precise syringe pump (11 Pico Plus, Harvard Apparatus, Cambridge, MA, USA) supplied the solution to the nozzle at a controllable flow rate. The nozzle-to-substrate distance was adjusted according to experimental requirements. The substrate was fixed on an XY stage such that the trajectories of electrosprayed patterns could be controlled.

Zinc acetate (ZnAc) aqueous solution was used as the electrosprayed solution in the experiments. The concentration of ZnAc in the solution was 4 wt%. The electrospray process was recorded by a CCD camera. The as-prepared samples were characterized with an optical microscope and a scanning electron microscope (SEM). Experimental data was measured and averaged from more than 10 samples.

## 3. Results and Discussion

In the conventional electrospray process, the nozzle-to-substrate distance is about 10–50 cm, and the diameter of the deposition area is usually larger than 5 cm, which is not suitable for the fabrication of micropatterns. In order to reduce the deposition area, the nozzle-to-substrate distance can be shortened to several millimeters. However, this tends to result in a continuous jet for a lowly conductive solution, or an unstable ejection for a highly conductive solution.

**Figure 2:**
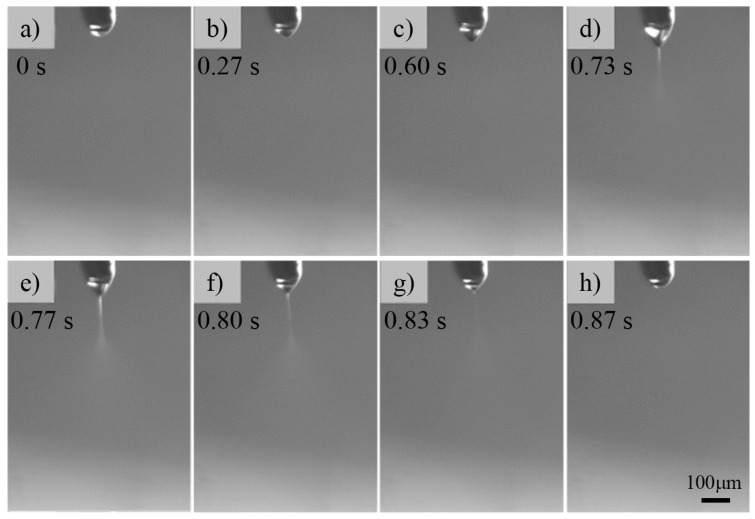
Discontinuous pulsed ejection of 4 wt% ZnAc aqueous solutions using a steel nozzle with an inner diameter of 60 μm and an outer diameter of 110 μm. (**a**–**c**) Solution pendant deformed by the applied voltage; (**d**,**e**) Liquid ejects from the nozzle; (**f**–**h**) The jet withdraws to the nozzle to form a hemispherical pendant. The nozzle-to-substrate distance was 2 mm, the applied voltage was 3 kV, and the flow rate of solution was 50 μL/h.

Figure 2 shows an unstable pulsed ejection of a 4 wt% ZnAc aqueous solution using a steel nozzle (inner diameter = 60 μm; outer diameter = 110 μm). The nozzle-to-substrate distance was 2 mm, the applied voltage was 3 kV, and the flow rate of solution was 50 μL/h. The applied voltage generated enough electric force to deform the solution pendant attached below the nozzle into a conical shape (Figure 2a–c). When the electric force overcame the surface tension, a jet was induced from the tip of the liquid pendant, as shown in Figure 2d,e. This jet atomized after traveling a short straight path and deposited on the substrate in the form of tiny droplets. Due to the high conductivity of the ZnAc aqueous solution, the ejection was not continuous, as the jet withdrew to the tip of the nozzle to form a hemispherical pendant (Figure 2f–h). Ejection was then repeated, thus representing a pulsed ejection mode.

**Figure 3:**
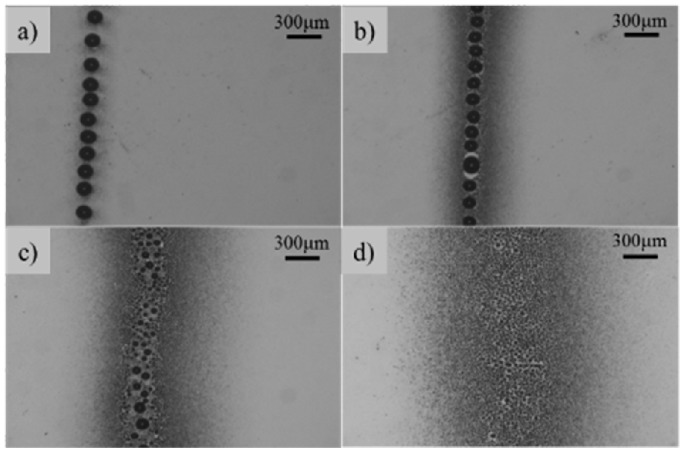
Electrosprayed patterns generated using the steel nozzle at a nozzle-to-substrate distance of (**a**) 1 mm; (**b**) 3 mm; (**c**) 5 mm; and (**d**) 5 mm. The flow rate was 50 μL/h for (**a**–**c**) and 100 μL/h for (**d**). The applied voltage was 3 kV.

Figure 3 shows the patterns deposited on the substrate at various nozzle-to-substrate distances. When the nozzle-to-substrate distance was set to 1 mm with an applied voltage of 3 kV and a flow rate of 50 μL/h, the electrospray process revealed a pulsed ejection. Due to the short nozzle-to-substrate distance, the jet did not have enough time to atomize, so the solution was discretely deposited on the substrate in the form of large droplets surrounded with satellite particles (Figure 3a). By increasing the nozzle-to-substrate distance to 3 mm (Figure 3b) and 5 mm (Figure 3c), more satellite particles and smaller droplets appeared. Figure 3d shows that uniform electrosprayed patterns could be generated at a nozzle-to-substrate distance of 5 mm, with an applied voltage of 3 kV and a flow rate of 100 μL/h. However, the width of the deposited pattern was larger than 1 mm and was not suitable for microscale fabrication.

**Figure 4:**
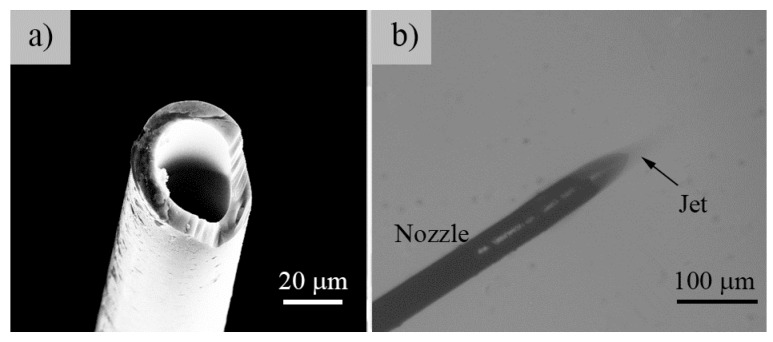
(**a**) SEM image of glass capillary nozzle. The inner diameter was about 30 μm; (**b**) Optical image of a stable continuous electrospraying process using a glass capillary nozzle. The applied voltage was 1.3 kV, the nozzle-to-substrate distance was 0.5 mm, and the flow rate was 100 μL/h.

During the electrospray process, adequate nozzle-to-substrate distance is required to allow the charged jet to atomize via the spraying process. Experimentally, this distance was comprehensively affected by the applied voltage, solution properties, nozzle shape, and substrate shape. Overall, an effective means to improve the electrospray performance with a short nozzle-to-substrate distance is to reduce the size of the charged jet emerging from the nozzle and to increase the Coulomb repulsion in the jet. Typically, Coulomb repulsion increases with increasing applied voltage. However, a high applied voltage would result in atmospherical discharge and interrupt the electrospray process, especially for high conductivity solution. Therefore, we developed a simple method to achieve continuous near-field electrospray using a tiny glass capillary nozzle with an inner/outer diameter of about tens of micron, the SEM image of which is depicted in Figure 4a. A nozzle with a smaller radius would produce a higher electric field intensity on the liquid meniscus and increase the electric force and Coulomb repulsion, which promotes the atomization of the charged jet. Generally, this tiny nozzle is introduced to achieve an intensified electric field that activates the electrospray and the thin charged liquid jet for atomization. Another benefit of a glass capillary nozzle is its insulating property to overcome the corona discharge at the nozzle tip under highly applied voltage. Figure 4b illustrates the continuous stable electrospray of the ZnAc solution with an applied voltage of 1.3 kV, a nozzle-to-substrate distance of 0.5 mm, and a flow rate of 100 μL/h. This stable ejection is beneficial for the fabrication of microscale atomized patterns.

**Figure 5:**
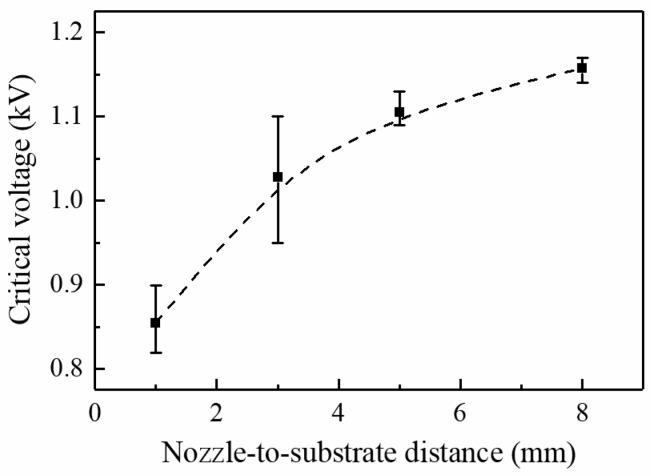
The effect of nozzle-to-substrate distance on critical voltage. The flow rate is 100 μL/h.

**Figure 6:**
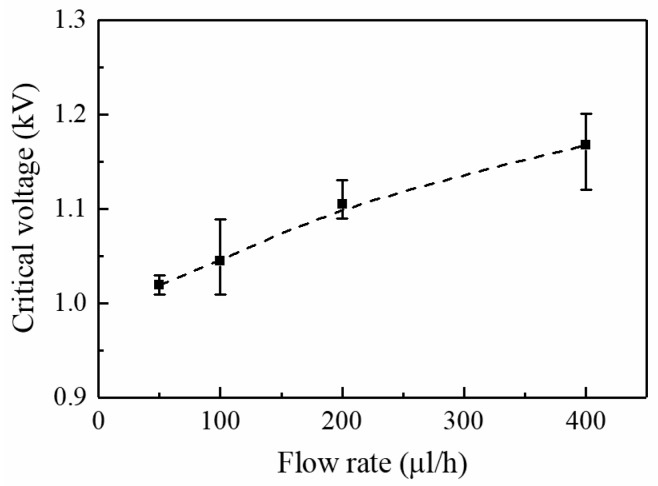
The effect of flow rate on critical voltage. The nozzle-to-substrate distance is 3 mm.

In the electrospray setup, the solution is pumped to the nozzle at certain flow rate, and it is subject to an external electric field generated from an applied voltage. Given a certain flow rate, cone-jet ejection occurs when the applied voltage reaches a corresponding critical value. Steady ejection can be maintained with the applied voltage remaining above the critical value at limited range [15,16]. The effects of nozzle-to-substrate distance and flow rate on the critical voltage for continuous near-field electrospray are investigated. Figure 5 shows the influence of the nozzle-to-substrate distance on the critical voltage at a fixed flow rate of 100 μL/h. The intensity of the electric field at the nozzle tip depends on the geometries of the nozzle, substrate, and nozzle-to-substrate distance. The electric field intensity reduces with increasing nozzle-to-substrate distance. As a result, critical voltage increases as nozzle-to-substrate distance increases to provide a sufficient electric field force for the electrospray process. The average critical voltages for nozzle-to-substrate distance of 1, 3, 5, and 8 mm are 0.86, 1.03, 1.11, and 1.16 kV, respectively. Figure 6 depicts the effect of the flow rate on the critical voltage at a fixed nozzle-to-substrate distance of 3 mm. Larger flow rate requires greater critical voltage to maintain a stable ejection, otherwise excess solution will cause the occurrence of dripping. The average critical voltages for a flow rate of 50, 100, 200, and 400 μL/h are 1.02, 1.05, 1.10, and 1.17 kV, respectively. A similar trend of the critical voltage versus the flow rate is also observed for traditional electrospray [17].

**Figure 7:**
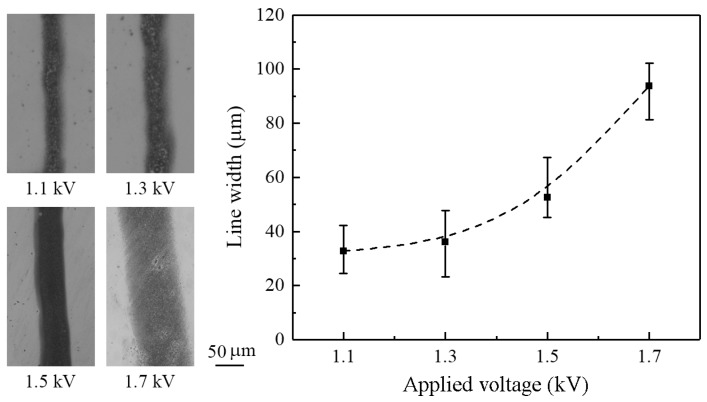
The effect of applied voltage on line width. The flow rate is 100 μL/h, and the nozzle-to-substrate distance is 0.5 mm.

Similar to the conventional electrospray process, the deposition of continuous near-field electrospraying can be adjusted by controlling operating parameters. The effect of applied voltage on the line width of electrosprayed patterns was evaluated, with the flow rate and nozzle-to-substrate distance set to 100 μL/h and 0.5 mm, respectively. The applied voltage affected the breakup of the charged liquid jet/droplet. Increasing the applied voltage led to a greater surface charge density on the jet and electric field, resulting in an increased electrostatic force. This increased electrostatic force induced a greater stretching force on the liquid and a stronger Coulombic repulsive force between the atomized droplets, resulting in a larger deposition area. As demonstrated in Figure 7, the line widths were 33, 36, 53, and 94 mm when the applied voltages were 1.1, 1.3, 1.5, and 1.7 kV, respectively.

**Figure 8:**
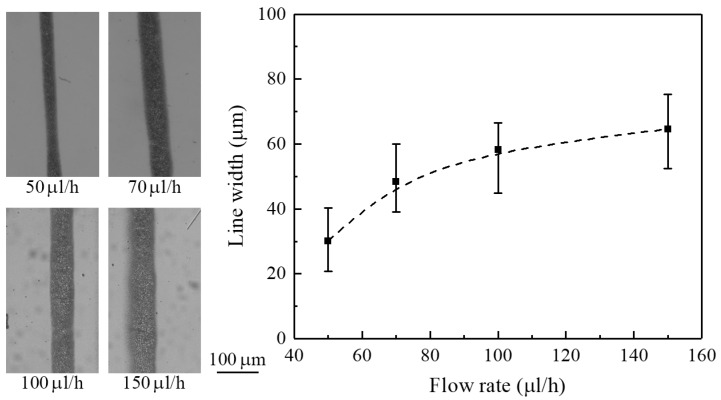
The effect of flow rate on line width. The applied voltage is 1.5 kV, and the nozzle-to-substrate distance is 0.5 mm.

The influence of flow rate on line width was investigated with an applied voltage of 1.5 kV and a nozzle-to-substrate distance of 0.5 mm. When the flow rate increased from 50 to 150 μL/h, the obtained line width increased from 30 to 65 μm, as shown in Figure 8. The increase in line width with increasing flow rate can be explained by the fact that increasing the flow rate contributes to the breakup of primary droplets and the formation of secondary/satellite droplets during atomization [18]. The droplet breakup occurred on the trajectory near the Taylor-cone, and the secondary/satellite droplets had high mobility and moved out of the electrospray stream, thus enlarging the line width.

**Figure 9:**
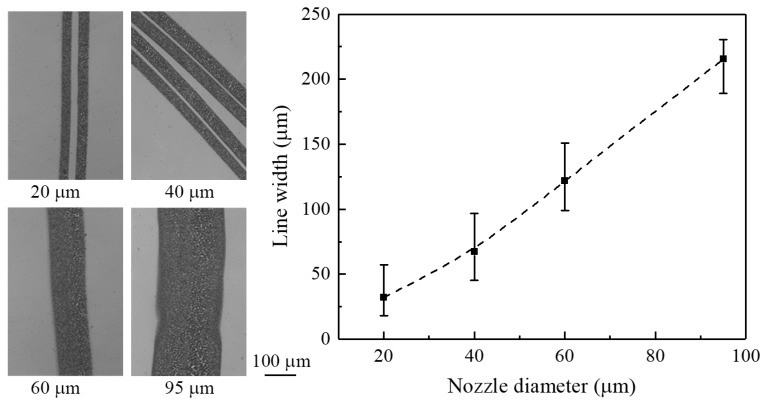
The effect of nozzle diameter on line width. The applied voltage is 1.5 kV, the nozzle-to-substrate distance is 0.5 mm, and the flow rate is 100 μL/h.

Figure 9 shows the effect of nozzle diameter on line width. The applied voltage is 1.5 kV, the nozzle-to-substrate distance is 0.5 mm, and the flow rate is 100 μL/h. It can be seen that the line width of patterns increases with the increase in nozzle inner diameter. The average line widths generated from nozzle diameters of 20, 40, 60, and 100 μm were 32, 67, 122, and 215 μm, respectively. Increasing the line width probably caused the diameter of the initial jet before atomization to grow with the inner diameter of the nozzle, thus leading to a larger deposition area. In addition, the ratios of the line width to the nozzle diameter were 1.6, 1.68, 2.03, and 2.26, which indicates that a nozzle with a small diameter is beneficial to the formation of small patterns.

Principally, the electrosprayed droplets are highly monodispersed in size distribution as they travel from the nozzle to the substrate. Among these, the primary droplets mainly distribute in the inner part of the electrospray zone, while the satellite droplets disperse to the outer part [18]. As a result, more liquid gathers at the central region than the edge region for deposition. In addition, solvent evaporation occurs on the surface of the liquid droplets. If the material reaching the substrate remains in liquid phase, i.e., as a droplet, the surface tension will make them merge together to form larger drops. Therefore, in the present work, the optical images of deposited patterns exhibit some irregularities, such as abnormality in material density and droplet size, as can be observed in Figure 3c,d and Figure 7, Figure 8 and Figure 9. However, uniformly solid particles may form if the solvent is fully evaporated before arriving at the substrate.

**Figure 10:**
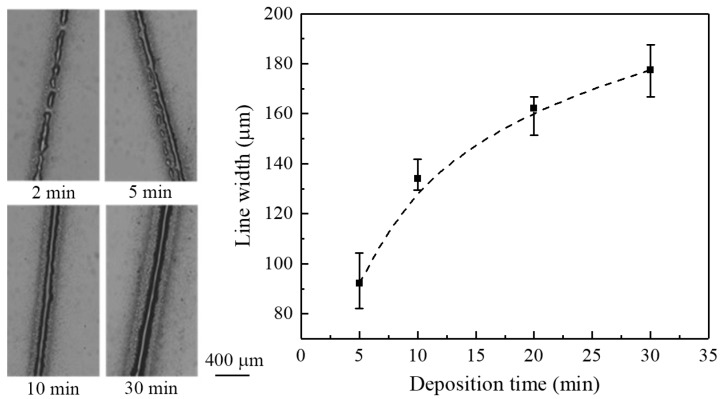
The effect of deposition time on line width. The nozzle-to-substrate distance is 0.5 mm, the applied voltage is 1.5 kV, and the flow rate is 100 μL/h.

To prepare continuous thin films, the effect of deposition time on the width of electrospray lines was investigated. Figure 10 shows the morphology and line width of thin films generated for various deposition times, with the nozzle-to-substrate distance, applied voltage, and flow rate fixed to 0.5 mm, 1.5 kV, and 100 μL/h, respectively. The electrosprayed line pattern reveals discrete irregular droplets in the early stage, i.e., 2 min, while a continuous track was generated when the deposition time was more than 5 min. Generally, the line width of an electrosprayed pattern increased with increasing deposition time. The average line width increased from 92.3 to 177.6 μm when the deposition time increased from 5 to 30 min. Furthermore, there were satellite droplets surrounding the line patterns. The amount of solution and charges on the substrate increased with deposition time, resulting in an increasing Coulombic repulsive force. Satellite droplets spread away from the electrospray axis [19], and the radical distance increases with the Coulombic repulsive force as well as the deposition time.

**Figure 11:**
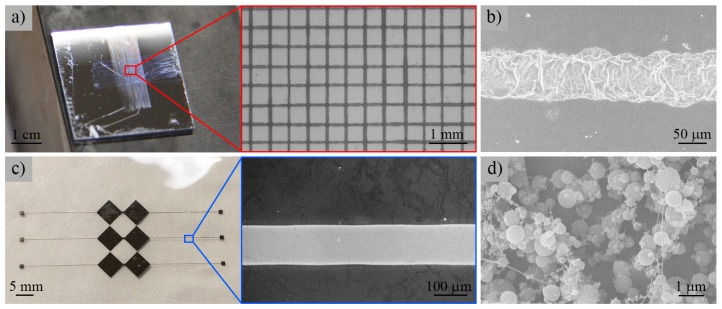
Various electrosprayed patterns. (**a**) ZnAc grid patterns deposited on a silicon substrate; (**b**) ZnO pattern generated by calcining the as-prepared ZnAc in air at 773 K for 30 min; (**c**) Silver pattern deposited on a glass substrate; (**d**) Chitosan nanoparticles electrosprayed on aluminum foil.

To demonstrate the controllability and continuity of our proposed process, an orderly ZnAc grid pattern was deposited onto a silicon substrate by controlling the trajectory of the motion stage, as shown in Figure 11a. To improve the continuity of thin films, polyethylene oxide (*M*_w_ = 300,000 g/mol) was added to the ZnAc aqueous solution with a concentration of 2 wt%. The applied voltage, nozzle-to-substrate distance, and flow rate were 1.2 kV, 0.5 mm, and 100 μL/h, respectively. The space between each line was 0.5 mm. The uneven spacing and line width was mainly due to the capacity of motion stage and the deviation of charged jet impacted from external perturbations [20]. Figure 11b illustrates a ZnO line pattern generated by calcining the as-prepared ZnAc in air at 773 K for 30 min. The continuous near-field electrospray allowed for the integration of ZnO micropatterns with a variety of device platforms, i.e., for sensor applications [21].

The electrospray process was compatible with a variety of functional materials. Figure 11c shows a conductive silver pattern electrosprayed on a glass substrate. The solution used was a commercial silver ink (TEC-IJ-040, InkTec Co., Ltd. Ansan, Korea), and the substrate was heated in air at 473 K during the electrospray process. The applied voltage was 1.0 kV, the nozzle-to-substrate distance was 0.5 mm, and the flow rate was 50 μL/h. Figure 11d illustrates the electrosprayed chitosan nanoparticles on aluminum foil. The solution was made from a compound of chitosan, acetic acid, and deionized water with concentrations of 4 wt%, 48 wt%, and 48 wt%, respectively. The applied voltage, nozzle-to-substrate distance, and flow rate during the experiment process were 3 kV, 3 mm, and 100 μL/h, respectively. The chitosan nanoparticles generated from continuous near-field electrospray had a morphology similar to that of conventional electrospray and may have potential biomedical applications [22].

Compared with the traditional electrospray process for micro-/nano-device fabrication, the continuous near-field electrospray is a simple and versatile direct-writing method for obtaining functional structures. The ability to electrospray functional materials for nano-sized particle deposition and thin-film coating at precise positions with controllable trajectories and specific patterns makes it useful for the low-cost integration of a variety of materials into devices. Potential applications may include micro-/nano-electronics, sensors, and biomedical engineering.

## 4. Conclusions

A continuous near-field electrospray process was investigated to generate microscale patterns using a shortened nozzle-to-substrate distance and a tiny glass capillary nozzle with a diameter of tens of micron. A steady and continuous ejection was achieved by this method. Micropatterns with line widths of sub-100 μm were generated. The influence of experimental parameters on the critical voltage was investigated. The increase in nozzle-to-substrate distance and flow rate increased the critical voltage of the initial stable jet ejection of the electrospray process. In addition, the line widths of electrosprayed patterns increased with the increases in applied voltage, flow rate, nozzle diameter, and deposition time. This study demonstrates a simple and promising method of the on-demand deposition of micro-/nano-patterns in the electrospray process that may be applied to the manufacture of electronic devices and biological systems.
